# Skin-lesion segmentation using boundary-aware segmentation network and classification based on a mixture of convolutional and transformer neural networks

**DOI:** 10.3389/fmed.2025.1524146

**Published:** 2025-03-10

**Authors:** Javaria Amin, Marium Azhar, Habiba Arshad, Amad Zafar, Seong-Han Kim

**Affiliations:** ^1^Rawalpindi Woman University, Rawalpindi, Pakistan; ^2^Department of Computer Science, University of Wah, Wah Cantt, Pakistan; ^3^Department of Artificial Intelligence and Robotics, Sejong University, Seoul, Republic of Korea

**Keywords:** skin lesion, compact convolution transformer, tokenizer, dermoscopy, hybrid loss, ResNet-34

## Abstract

**Background:**

Skin cancer is one of the most prevalent cancers worldwide. In the clinical domain, skin lesions such as melanoma detection are still a challenge due to occlusions, poor contrast, poor image quality, and similarities between skin lesions. Deep-/machine-learning methods are used for the early, accurate, and efficient detection of skin lesions. Therefore, we propose a boundary-aware segmentation network (BASNet) model comprising prediction and residual refinement modules.

**Materials and methods:**

The prediction module works like a U-Net and is densely supervised by an encoder and decoder. A hybrid loss function is used, which has the potential to help in the clinical domain of dermatology. BASNet handles these challenges by providing robust outcomes, even in suboptimal imaging environments. This leads to accurate early diagnosis, improved treatment outcomes, and efficient clinical workflows. We further propose a compact convolutional transformer model (CCTM) based on convolution and transformers for classification. This was designed on a selected number of layers and hyperparameters having two convolutions, two transformers, 64 projection dimensions, tokenizer, position embedding, sequence pooling, MLP, 64 batch size, two heads, 0.1 stochastic depth, 0.001 learning rate, 0.0001 weight decay, and 100 epochs.

**Results:**

The CCTM model was evaluated on six skin-lesion datasets, namely MED-NODE, PH2, ISIC-2019, ISIC-2020, HAM10000, and DermNet datasets, achieving over 98% accuracy.

**Conclusion:**

The proposed model holds significant potential in the clinical domain. Its ability to combine local feature extraction and global context understanding makes it ideal for tasks like medical image analysis and disease diagnosis.

## 1 Introduction

The skin is a major part of the human body and consists of the epidermis, dermis, lymphatic vessels, muscles, blood vessels, subcutaneous tissue, and nerves (1). Apart from protecting the whole body and various organs against external invasions such as chemical damage, the skin can also prevent adventitious viruses (2). Regardless of its protective and barrier functions, the skin is destructible; it tends to be affected by a diversity of genetic and external factors. Deterioration of lipids in the epidermis can be prevented by using liquids to improve the barrier features of the skin. Fungal development on the skin owing to allergic reactions, hidden bacteria, degradation of skin texture due to microbial reactions, and pigment creation can lead to various skin diseases (3). Skin diseases are considered chronic and may infrequently propagate into malicious tissues. Skin diseases must be treated promptly to restrict their growth and propagation (4). In recent years, imaging-technology-based research to identify the effects of various skin diseases has been in high demand. In most cases, different skin diseases may have similar appearances, making early detection of skin diseases difficult. Owing to the lack of contrast between adjacent tissues, predicting the type of skin lesion is also challenging. Other systems are unable to manage environmental- and texture-based variations in the input image, which is a significant concern because environmental and lighting conditions cannot always be controlled. The sheer number of skin diseases, combined with the difficulties caused by diverse environments and limited datasets, make skin disease classification a significant challenge (5). Computer-aided diagnostic techniques are preferred to classify skin diseases reliably and efficiently, assisting in medication prescription (6). Diseased growth propagation is assessed using a grey-level co-occurrence vector. To improve medication and minimize treatment costs, accurate diagnosis is essential to thoroughly assess abnormalities.

Owing to the limited and false distribution of experienced dermatologists, effective and efficient diagnosis using data-driven approaches is required as skin diseases are spreading in various shapes. The growing trend towards photonics-based and laser medical technology has made accurate and robust diagnosis of skin diseases feasible. However, these treatments are expensive and have limited applications. To address this issue, researchers are in search of more robust solutions, and convolutional neural networks (CNN) have been considered (7, 8). The major constraint in detecting skin diseases using CNNs is that they tend to learn and represent the bias inherent in the training data (9). For instance, the diagnostic accuracy of lesions on light skin is higher than on dark skin. This is because there may have been insufficient dark skin samples with the same lesion in the training set, or the image markers of protective factors and disease-affected regions may have an inherent correlation.

Approaches based on deep learning (DL) are considered more efficient than these techniques in classifying diseased parts from images in a dataset (6, 10–12). The growing need in healthcare diagnosis is to detect abnormalities precisely and to classify the category of disease from various types of biomedical images, such as magnetic resonance imaging, positron emission tomography, X-ray, and computed tomography scan data in the form of signals, that is, electroencephalogram (EEG), electrocardiogram (ECG), and electromyography (EMG) (13–19). Better treatment of patients according to the type of disease can be achieved by precisely identifying the disease category. Critical problems can be solved using DL models, enabling them to automatically detect input features. The inferred data can be obtained through deep-learning-based models that use unexposed data patterns to identify data features. Even DL models with low computational costs can result in optimal efficiency.

Owing to the challenges in previous studies, the key objectives of the proposed methodology are as follows:

• To develop an optimized deep-learning model for skin lesion segmentation by integrating prediction and refinement modules for enhanced accuracy.
• To utilize a hybrid loss function (SSIM, IoU, BCE) for improved segmentation performance by preserving structural details and optimizing overlap measures.
• To propose a compact convolutional transformer model (CCTM) with optimized layers and hyperparameters for efficient and accurate classification of skin lesions.
• To evaluate the performance of the proposed model against existing state-of-the-art methods in terms of segmentation accuracy, computational efficiency, and classification effectiveness.

To achieve the abovementioned objectives, a deep learning-based solution is proposed, whose major contributions are as follows:

• The proposed BASNet model comprises prediction and refinement modules for skin-lesion segmentation. It is trained from scratch on hybrid loss, which is a fusion of structural similarity, intersection over union, and binary cross-entropy.
• The prediction module works like a U-Net and is densely supervised by the encoder and the decoder. The encoder contains an input convolution layer and six stages. Four stages were adopted from ResNet-34 and retained from the basic residual block. The bridge and decoder use three convolution layers and side outputs, respectively. This module generates seven probability maps for segmentation, of which the last map is considered the final output.
• The module of residual refinement is used to further refine the map of the final output through residual learning among ground truth courses and maps.
• A compact convolutional transformer model (CCTM) is proposed for a selected number of layers and hyperparameters with two convolutional transformers, two transformers, 64 projection dimensions, 64 batch sizes, two heads, 0.1 stochastic depth, 0.001 learning rates, 0.0001 weight decays, and 100 epochs that provide excellent outcomes for classification.

The remainder of the paper is organized as follows. Related work is described in Section 2. The proposed methodology, with its various steps for identifying and classifying skin diseases, is discussed in Section 3. In Section 4, the quantitative and qualitative results are discussed, and Section 5 provides a brief conclusion of the proposed research work and its future perspectives.

## 2 Related work

Recently, researchers have introduced extensive methods to detect and classify skin diseases in their early stages using computer vision, machine learning, pattern recognition, deep CNN models, and artificial intelligence (20, 21). Rajeswar et al. (22) used the wolf antlion neural network (WALNN) to classify skin melanomas using magnetic resonance imaging data. A hybrid algorithm was introduced for feature selection. WALNN was compared with established methodologies, such as Cuckoo search-based SVM, decision tree, and CNN on the ISIC skin-lesion dataset. It improved sensitivity, specificity, recall, accuracy, and precision, and accurately identified skin melanoma. Khan et al. (23) presented a method that uses mobile health units to collect skin data using a multimodal data fusion for skin-lesion detection. This system uses a hybrid approach for lesion segmentation by combining two CNN models. The HAM10000 dataset was used to train the CNN model for lesion classification. A summation discriminant correlation testing approach was applied to combine features from the two connected layers. A feature selection method was introduced to prevent feature redundancy. An ultimate machine-learning classifier was applied to classify the selected features with remarkable outcomes, in contrast to those of the traditional methods. Renkai et al. (24) suggested that dermoscopy is highly useful in diagnosing skin diseases, particularly skin lesions. Automatic skin-lesion segmentation is crucial for accurate diagnoses. Although U-Net models are commonly used for segmentation tasks, they have limitations in terms of spatial dependence and remote interactions. Transformers are emerging as alternatives; however, they require large amounts of data and significant computational resources. To address these issues, HorUNet, along with a multilevel dimensional fusion mechanism, was introduced. Extensive experiments were performed using private and publicly available ISIC2017, ISIC2018, and PH datasets. Ordinary convolution, which fails to exhibit spatial dependence or remote interaction, was used in the U-Net model. Transformers are becoming a popular alternative. They require large amounts of data and huge computational resources, making them less practical for clinical medical problems. Rahman et al. (25) suggested that existing deep-learning-based schemes do not explore concurrent multi-image comparative methods. To improve the diagnosis of melanoma, a feature fusion method that integrates patient-related information was proposed. The introduced multiple-kernel self-attention segment provides an optimal overview of the extracted features, which are combined using a contextual feature fusion approach from various images into a distinct feature matrix. The introduced contextual-learning scheme significantly improved performance. Akilandasowmya et al. (26) split the deep hidden features to ensure accurate predictions and, applied a harmony search technique to optimize the features (25) and reduce data size. They also used ensemble classifiers for early disease diagnosis. Results on ISIC-2019 and Kaggle skin-lesion datasets showed considerable improvement compared with traditional methods. ul haq et al. (27) introduced a hybrid-equilibrium Aquila optimization method using random forests and ensemble support vector kernels. The HAM10000 dataset, with enhanced image resolution after removing intrusions and noise, was used to test the model. The framework subcategorized the segmented images into five classes based on the feature properties. This approach achieved a high performance rate with an accuracy of approximately 97.4%. Kalpana et al. (28) suggested a combination model combining CNN and U-Net models to create an automated system capable of recognizing skin lesions in biomedical dermoscopic images. CNN was used to classify segmented images into multiple classes. The system was designed to handle biomedical image data, ensuring accurate and fast recognition of skin lesions to enhance the effectiveness of the DL-based approach in treating various illnesses. Two optimizers with distinct batch sizes were used to optimize the proposed scheme. Anand et al. (29) presented a cutting-edge technique called SSD-KD that integrates disparate information into a general knowledge distillation technique for skin disease classification. This methodology combined the current knowledge distillation research by developing intra-instances to represent relational features. The apprentice model captured more information than the instructor model using weighted softened outputs. The condensed MobileNetV2 classified eight distinct skin illnesses with an accuracy of up to 85%. This is the first application of deep knowledge distillation to a large-scale dermoscopy database for multidisease categorization. The SSD-KD method for skin disease classification, although effective, is computationally intensive and may not be suitable for all portable devices. However, its performance depends on the training data quality, which can limit its generalizability. The complexity of the dual relational knowledge distillation architecture adds to implementation challenges. Table 1 provides a summary of existing techniques.

**TABLE 1:** Existing methods for skin-lesion detection.

References	Methods	Findings	Limitations
Khan et al. (23)	Mobile health units for data collection; multiple modal data fusion approach	Effective segmentation and classification of skin lesions with improved results	Requires mobile data collection; complex feature selection and fusion
Wu et al. (24)	HorUNet with multilevel dimensional fusion mechanism	Improved segmentation accuracy for skin lesions using ISIC2017, ISIC2018, PH datasets	High computational requirements for large datasets
Rahman et al. (25)	Features fusion method	98.3% accuracy and 95.7% AUC score on ISIC-2020 dataset	Limited exploration of concurrent multi-image comparison methods beyond melanoma
Akilandasowmya et al. (26)	Deep hidden features, ensemble classifier	Improved early detection results using ISIC-2019 and Kaggle skin-lesion data	Harmony search method complexity for feature optimization
Vidhyalakshmi and Kanchana (48)	Binary butterfly optimization algorithm (BBOA) and DCNN for skin disease categorization	Automated categorization with high prognosis accuracy	Limited applicability beyond specific skin disease types
ul haq et al. (27)	Hybrid equilibrium Aquila optimization with random forests and ensemble support vector kernels	Achieved approximately 97.4% accuracy for skin disease categorization using HAM10000 dataset	Dataset quality dependent due to requirement for enhanced image resolution
Kalpana et al. (28)	Combination of CNN and U-Net models for lesion segmentation and classification	Effective and fast recognition of skin lesions	Optimization complexity with two distinct batch size optimizers
Anand et al. (29)	SSD-KD integrating knowledge distillation (KD) for skin disease classification	85% accuracy for eight skin illnesses with condensed MobileNetV2 using ISIC-2019	Limited generalizability of KD technique for diverse datasets

Table 2 summarizes the datasets used in the proposed method.

**TABLE 2:** Overview of datasets.

Dataset name	Images	Class names	Resolution of images
PH2	200	Atypical Nevi, Common Nevi, Melanoma	768 × 560
MED-NODE	170	Benign Nevus, Melanoma	Not specified
ISIC 2016	1,279	Background, Skin Lesion	576 × 786 to 2,848 × 4,288
ISIC 2017	2,750	Nevus, Seborrheic Keratosis, Melanoma	556 × 679 to 4,499 × 6,748
ISIC 2018	10,015	Skin Cancer, Pigment Network, Globule, Milia-like Cyst, Negative Network, Streaks	Not specified
DermNet	19,500	23 Categories (e.g., Eczema, Seborrheic Keratoses, Poison Ivy, Acne, etc.)	Varies, generally low resolution

To overcome the existing challenges, the proposed BASNet effectively generalizes features learned in a multiscale and dense supervision module, allowing the capture of fine-grained and global contexts regarding the details of the boundary. A residual network was combined to extract semantic high-level features with boundary refinement to improve the accuracy of boundary predictions. Hybrid loss includes structural similarity and boundary-aware losses, which aid the model in focusing on fine boundaries and shapes to improve performance on unseen and diverse data. By combining the advantages of transformers with those of convolutional layers, the proposed CCTM performed well in terms of generalization. Convolutional layers are prone to significant inductive biases when learning local patterns, such as edges and textures. This improves the generalization of the model on small datasets or sparse data. The model can comprehend complicated spatial linkages successfully with small amounts of data, which enhances the performance of various applications and datasets.

## 3 Materials and methods

**FIGURE 1:**
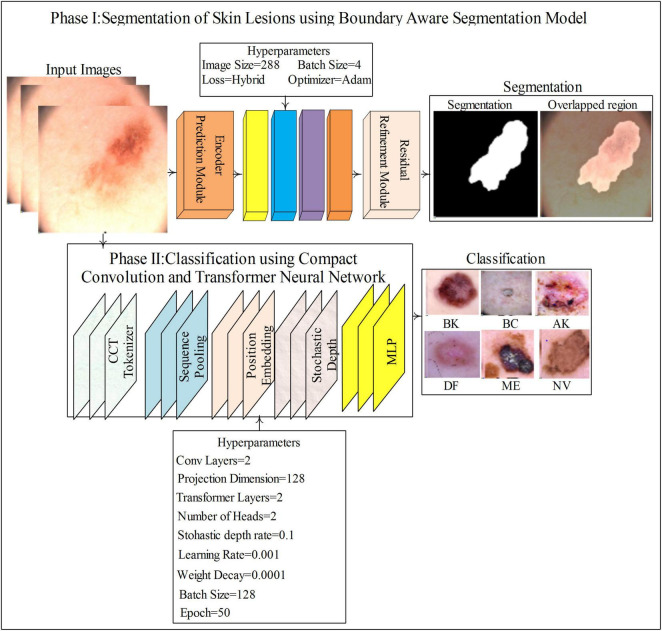
Proposed method for skin lesions detection.

In this study, two novel models are proposed for the segmentation and classification of skin lesions. BASNet is fine-tuned to segment skin lesions in poorly contrasted, illuminated, and hair dermoscopy images of the skin. The compact transformer model, which is a mixture of convolutional and transformer models, is proposed for classification. The proposed method steps are illustrated in Figure 1.

**FIGURE 2:**
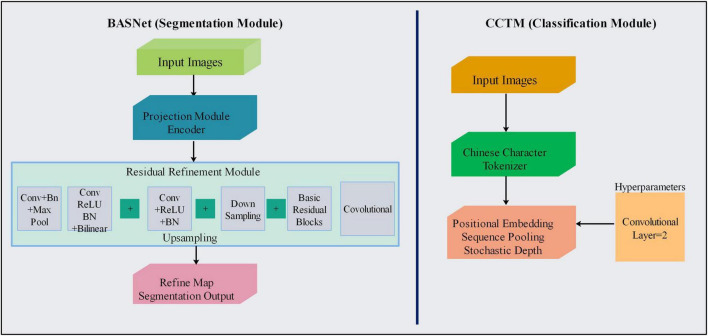
Flowchart of proposed models for segmenting and classifying skin lesions

The classification model comprises compact convolutional and transformer models (Figure 1), consisting of a tokenizer, position embedding, sequence pooling, stochastic depth, and MLP to classify SL. Figure 2 shows the segmentation and classification model architectures in detail.

### 3.1 Proposed boundary-aware segmentation model

**FIGURE 3:**
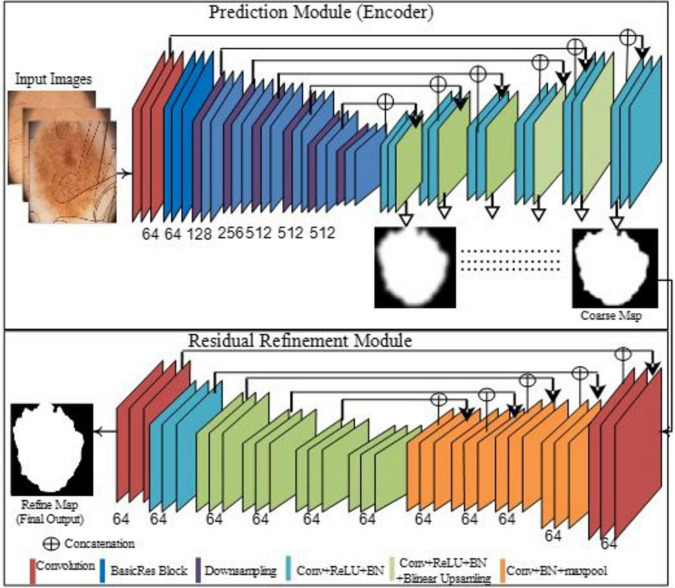
Proposed segmentation model of the skin lesions.

BASNet contains prediction and residual multiscale refinement modules and a refined network for prediction and hybrid losses (30). The refined prediction model comprises a dense encoder/decoder supervised model. The prediction module consists of an encoder and decoder similar to that of U-Net. The encoder section contains an input layer of convolutional layers and six stages, where the first four are taken from the ResNet-34 and the remaining are from the primary residual blocks. The first convolution layer and pool of ResNet-34 are skipped, and four blocks are extracted. The decoder and bridge use three convolutional layers with side outputs. This module generated seven probability maps for segmentation during training, the last of which is the output layer. The objective of the refinement module—based on a residual block—is to refine noisy and blurry boundaries on the segmentation maps produced through prediction. This model comprises four stages, each consisting of a convolutional block. Finally, the coarse and residual maps produce a more refined output map, as shown in Figure 3.

Hybrid loss helps the network learn in a hierarchy, such as the patch, pixel, and map levels, as defined in Equation 1 (31).


(1) $$\mathrm{Sum}\,\mathrm{of}\,\mathrm{output}\,\left(\mathrm{SOO}\right)=\sum_{n=1}^{N}\alpha_{n^{l^{\left(n\right)}}}$$

where SOO is the sum of the outputs, N is the total output, n is a single instance of outputs, l^(n)^ is a loss of n outputs, and α_n_ is the weight of each loss. This model has eight outputs: seven related to the prediction model and one for refinement.

Clearer and higher-quality segmentation boundaries were obtained using hybrid loss, expressed as Equation 2.


(2) l(n)=l(n)bce+l(n)ssim+l(n)iou

where l^(n)^bce, l^(n)^ssim, l^(n)^iou are the BCE, SSIM, and IoU losses, respectively. Mathematically, the BCE is defined as Equation 3.


(3) $$l_{\mathrm{bce}=-\sum_{\left(r,c\right)}\left[M\,\left(r,c\right)\,\log\,\left(P\,\left(r,c\right)\right)+\left(1-M\,\left(r,c\right)\right)\,\log\,\left(1-P\,\left(r,c\right)\right)\right]}$$

where M(r,c) ∈ 0,1 is the pixel (r, c) of the annotated mask, and P (r, c) is the probability of the predicted pixel. SSIM loss captures the structural information of the image. Let x = x_j_: j = 1,…,N^2^ and y = y_j_: j = 1,…,N^2^ represent the pixel values of the patch size in N = N region cropped from p probability map and m mask, respectively. The SSIM of *x* and *y* are expressed as Equation 4.


(4) lSSIM=1-(2⁢mx⁢my+Cov1)⁢(2⁢σxy+Cov2)(m2x+m2y+Cov1)⁢(σ2x+σ2y+Cov2)

where m_x_, m_y_, σ_x_, and σ_y_ represent the mean and standard deviation of x and y, respectively. σ_xy_ denotes covariance. The values of the Cov_1_ and Cov_2_(0.01^2^ and 0.03^2^) were used to prevent division by 0.

The intersection over union (IoU) matrix was used to compute the similarity using Equation 5.


(5) $$l_{\mathrm{IoU}}=1-\frac{\sum_{r=1}^{H}\sum_{c=1}^{W}p\,\left(r,c\right)\,M\,\left(r,c\right)}{\sum_{r=1}^{H}\sum_{c=1}^{W}\left[p\,\left(r,c\right)+M\,\left(r,c\right)-p\,\left(r,c\right)\,M\,\left(r,c\right)\right]}$$

where M(r,c)ϵ0,1 is pixel (r, c) and p (r, c) is predicted pixel probability.

The BCE loss was used to compute pixel-level values. The neighborhood of the labels was not considered, and foreground and background pixels were equally weighted, which helps with convergence points on pixels and guarantees the best local optima. The SSIM loss was used for patch-level information around the pixels of the local neighborhood. Higher weight values were assigned to the pixels located in the region of the transitional buffer between the foreground and background pixels that are similar or higher than the region of the foreground. The background loss was not applied in training until the background pixels were closer to the mask, where the loss dropped rapidly to 1–0.

The values of m_x_, σ_xy_, m_x_m_y_, and σ^2^y in SSIM_*loss*_ in Equation 4 were 0 in the region of the background. Therefore, the SSIM value was computed using Equation 6.


(6) lbackgroundSSIM=1-Cov1⁢Cov2(mx2+Cov1)⁢(σx2+Cov2)

where Cov_1_ = 0.01^2^ and Cov_2_ = 0.03^2^, when the prediction of x was close to zero.

IoU was used to compute map-level information. If a large region is included in the IoU, the model trained on the IoU loss emphasizes a large region of the foreground and generates homogeneous and whiter probabilities in these areas. However, this model produced a false-negative region in finer structures. To obtain the advantages of the three losses—BCE, IoU, and SSIM—the hybrid loss was formulated. BCE was used to maintain the gradient among all pixel values, and IoU focused on the foreground pixels.

### 3.2 Classification of the skin lesions based on the proposed mixture of convolutional and transformer mode

**FIGURE 4:**
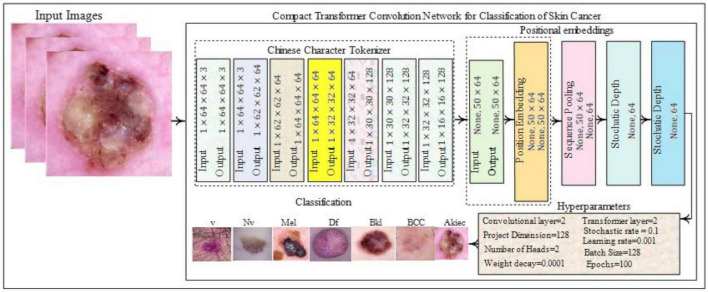
Proposed classification model.

The proposed CCTM model for skin-lesion classification is shown in Figure 4. The model uses a tokenizer to process input images. The vision-transformer (ViT) model was applied to organize all images into a uniform non-overlapping patch, removing information related to the boundaries between distinct patches, which is vital for effectively exploiting the local information. Convolution works well in extracting local information. The input images were processed using a tokenizer. The ViT model divided images into non-overlapping uniform patches to reduce the information at the boundaries between different types of patches. This is vital for neural networks to effectively exploit local information. The convolution kernel effectively exploits the local information; thus, the convolution in the mini-model generates patches. Positional embedding in CCT is optional, and sequence or attention pooling was added. In the ViT model, the mapping of features related to the token of the class is pooled and used subsequently for skin-lesion classification. Stochastic depth is a regularization method of randomly dropping a set of layers. This is similar to dropout but operates on a block of layers as compared to separate nodes that are included in the layer. Stochastic depth is used before the residual blocks of the transformer section of the encoder. Finally, the encoder section of the transformer is weighted and fed to the final specific layer for skin-lesion classification.

## 4 Experimental results and discussion

To evaluate the effectiveness of the segmentation method, four publicly available datasets were used: PH2 (32), ISIC 2016 (32), 2017 (33), and 2018 (34). Five publicly available datasets were used to evaluate the performance of the classification method: PH2 (32), HAM10000 (35), ISIC 2019 (36), MED-NODE (37), and DermNet (accessed on 9 November 2022).^1^ The entire datasets were divided into 0.4 hold-out validation, in which 60% of the data was used for training, 20% for validation, and 20% for testing. The proposed method was implemented on a Desktop-T20JD6R, 12th Generation, Core i7-12700K, processor 3.60 GHz, RAM 32.0 GB, graphics card NVIDIA RTX A4000 with a Windows-11 operating system.

### 4.1 Segmentation and classification datasets

In this section, the datasets used for segmentation and classification are discussed. The details of the segmentation datasets are as follows.

*PH2:* The PH2 dataset contained 200 images of melanocytic lesions categorized as atypical nevi, common nevi, and melanomas. Each image was verified by expert dermatologists who manually segmented skin lesions for clinical analysis. This database is valuable for evaluating and validating computer-based segmentation and classification algorithms for melanoma diagnosis.

*ISIC 2016:* This dermoscopy dataset is categorized into two classes named “background” and “skin lesion.” The anatomical region “full body” skin is considered.

*ISIC 2017:* The size of the training and validation data was 2000/150 whereas the test data size was approximately 600.

*ISIC 2018:* A large-scale dataset published by ISIC containing 10,015 dermoscopic images with skin lesions annotated with seven classes of skin diseases such as skin cancer, pigment network, globule, and others: milia-like cysts, negative networks, and streaks. These classes were used to detect various skin diseases. The dataset was used for instances and semantic segmentation.

The details of classification datasets are as follows:

*HAM10000:* The HAM10000 dataset comprised a considerable group of multisource dermoscopic images containing colored skin diseases. Diverse populations and acquisition methods were used to collect 10,015 images. The dataset included seven generic classes of pigmented lesions, selected for simplicity and practical clinical relevance. The dataset was meticulously cleaned and standardized to ensure high quality using manual screening to exclude specific attributes and ensure appropriate color reproduction.

*ISIC 2019*: consisted of 19,424 dermoscopic images captured over 16 years using high-resolution cameras. These images comprised approximately 11 diagnostic groups. The dataset may become more disabled in medical practice by relating each captured image to the age and sex of the patient and the position of the lesion.

*MED-NODE:* This is a non-dermoscopic skin-lesion dataset consisting of two classes: benign nevi with 100 images and melanoma lesions with 70 images.

*DermNet:* Consisted of 19,500 images with three RGB channels and 23 distinct categories of skin diseases, such as eczema, borrheic keratoses, poison ivy, acne, vascular tumors, tinea ringworm, psoriasis, melanoma, and bullous disease. However, the images were mostly of low resolution.

The datasets had limitations such as data imbalance, low resolution, and poor contrast. The data were augmented through horizontal and vertical flipping to increase the number of images. Hybrid loss and residual refinements were applied to BASNet to handle occlusion and poor contrast images.

### 4.2 Experiment 1: segmentation of the skin lesions using the proposed BASNet model

**FIGURE 5:**
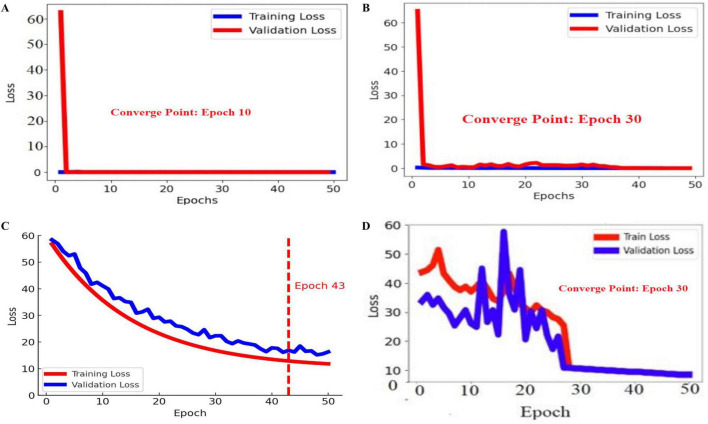
Training and validation losses of the proposed BASNet: **(A)** PH2, **(B)** ISIC 2016, **(C)** ISIC 2017, and **(D)** ISIC 2018.

In this experiment, the BASNet Model was trained on the optimal hyperparameters that are shown in Figure 5.

**FIGURE 6:**
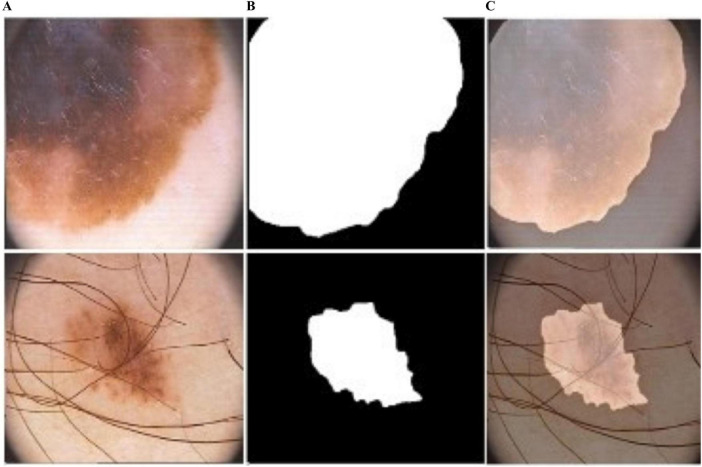
Predicted segmentation outcomes from ISIC-2017: **(A)** input **(B)** predicted masks, and **(C)** overlapped predicted output.

**FIGURE 7:**
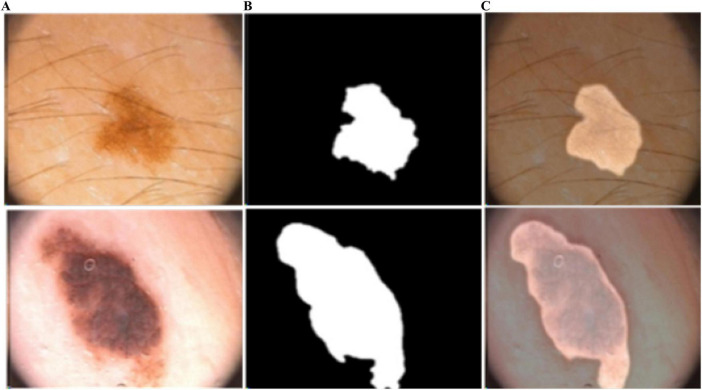
Predicted segmentation outcomes from PH2: **(A)** input, **(B)** predicted masks, and **(C)** overlapped predicted output.

**FIGURE 8:**
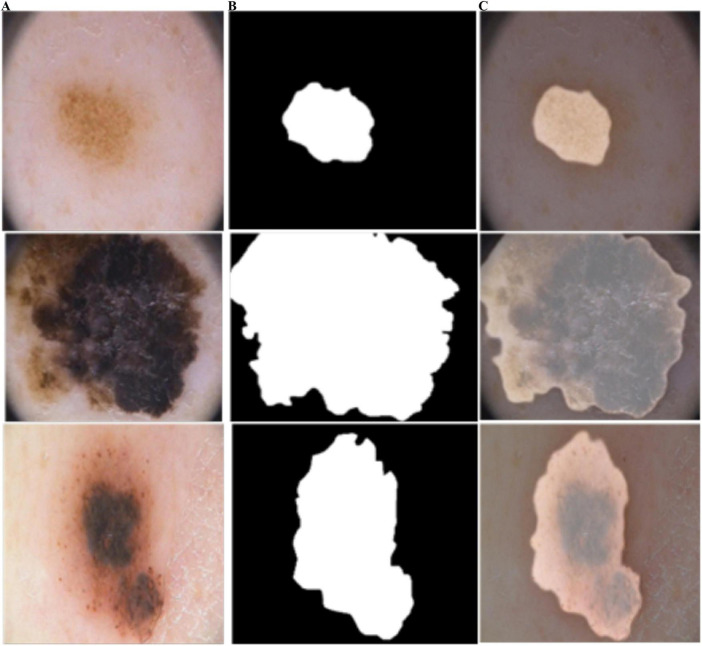
Predicted segmentation outcomes from ISIC-2018: **(A)** input, **(B)** predicted masks, and **(C)** overlapped predicted output.

**FIGURE 9:**
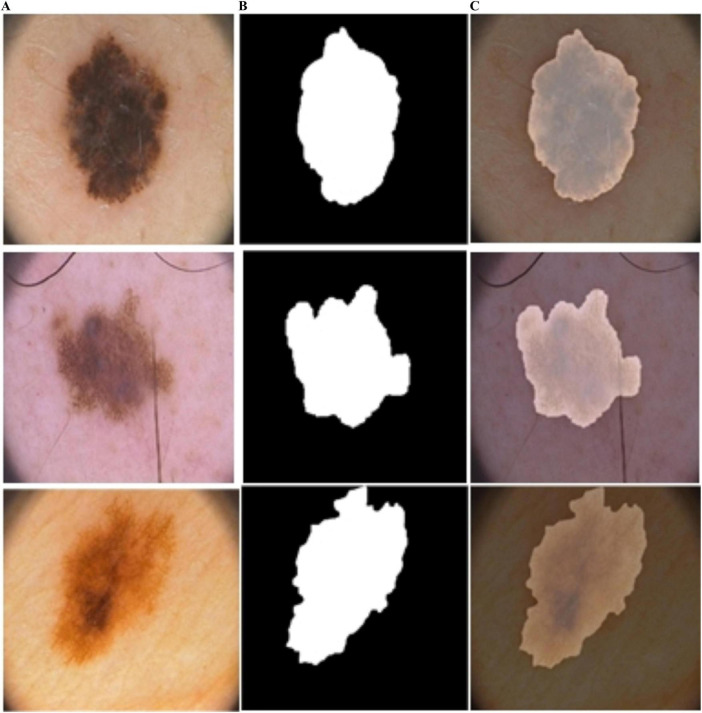
Predicted segmentation results from ISIC-2016: **(A)** input, **(B)** predicted masks, and **(C)** overlapped predicted output.

In Figure 4, the blue and red lines represent the training and validation losses, respectively. On the PH2 dataset, the training/validation loss was stable at the initial epochs, whereas on the ISIC [2016, 2017, and 2018] datasets, the training/validation curves were stable after 30 epochs. The segmentation model results are shown in Figures 6–9.

**FIGURE 10:**
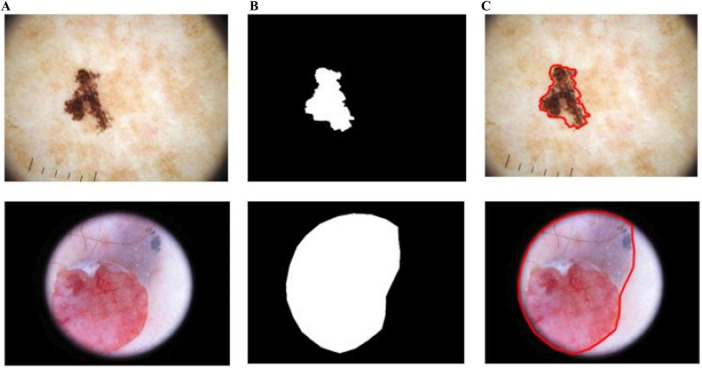
Segmentation results of BASNet: **(A)** input, **(B)** segmentation, and **(C)** skin-lesion boundaries.

As shown in Figure 10, BASNet segments the skin-lesion boundaries more accurately, even with illumination and lighting effects. The computed segmentation results are given in Table 3.

**TABLE 3:** Segmentation outcomes of the proposed BASNet model.

Segmentation datasets	Testing	Validation	Training	Average (%)		Confidence interval
				**IoU**	**Dice score**	
PH2	✓			0.96	0.98	[0.9383–0.9817]
		✓		0.96	0.98	[0.9383–0.9817]
			✓	0.95	0.98	[0.9283–0.9717]
ISIC 2016	✓			0.89	0.96	[0.8683–0.9117]
		✓		0.87	0.95	[0.8483–0.8917]
			✓	0.88	0.97	[0.8583–0.9017]
ISIC 2018	✓			0.94	0.97	[0.9183–0.9617]
		✓		0.93	0.96	[0.9083–0.9517]
			✓	0.96	0.98	[0.9383–0.9817]
ISIC 2017	✓			0.97	0.98	[0.9483–0.9917]
		✓		0.95	0.97	[0.9283–0.9717]
			✓	0.94	0.96	[0.9183–0.9617]

Table 3 shows the evaluation of the segmentation model using four dermoscopy datasets, PH2, ISIC 2016, ISIC 2018, and ISIC 2017. During training, the average IoU and Dice scores were 0.95 and 0.98 on PH2; 0.88 and 0.97 on ISIC 2016; 0.96 and 0.98 on ISIC 2018, and 0.94 and 0.96 on ISIC 2017 datasets, respectively. Similarly, in the validation stage, the results in terms of IoU and Dice scores were 0.96 and 0.98 on PH2; 0.87 and 0.95 on ISIC 2016; 0.93 and 0.96 on ISIC 2018, and 0.95 and 0.97 on ISIC 2017 datasets, respectively. Finally, in the testing stage, the results were 0.95 and 0.98 on PH2; 0.88 and 0.97 on ISIC 2016; 0.96 and 0.98 on ISIC 2018, and 0.94 and 0.96 on ISIC 2017 datasets, respectively.

The confidence intervals demonstrate high consistency and reliability across datasets. Most intervals fall within narrow ranges, indicating stable performance. For instance, several scores show a confidence interval of [0.9383, 0.9817], reflecting a robust and precise estimation. Similarly, intervals such as [0.9283, 0.9717] and [0.9183, 0.9617] suggest minimal variability, while slightly wider intervals like [0.8683, 0.9117] and [0.8483, 0.8917] indicate reduced but still consistent performance. Notably, the highest confidence interval [0.9483, 0.9917] showcases the peak reliability within the dataset. Overall, the reported intervals confirm a high level of accuracy and confidence. BASNet was trained for 50 epochs; the training time of each epoch is presented in Table 4.

**TABLE 4:** Comparison of training time on HAM-10000 dataset.

Model	Total epochs	Time of each epoch
BASNet model	50	4 min
Yaseliani et al. (49)		17.46 s

Table 4 shows the training times of BASNet and the existing method on the HAM-10000 dataset. The input image size is 288 × 288. This model takes four minutes on each epoch. However, the existing method is trained for 50 epochs with 240 × 240 input image size, taking 17.46 sec on each epoch. The difference in time is due to the image size. The outcomes are shown in Table 5.

**TABLE 5:** Results of proposed BASNet model compared to the existing approaches.

References	Year	Datasets	Results (%)
Giotis et al. (38)	2024	PH2	0.96
Hong et al. (42)	2023		0.96
Srikanteswara and Ramachandra (41)	2023		0.82
Nampalle et al. (40)	2022		0.92
Mustafa et al. (50)	2025		0.93
Proposed model			0.98
Giotis et al. (38)	2024	ISIC-2018	0.96
Nampalle et al. (40)	2022		0.89
Venugopal et al. (51)	2023		0.97
Proposed model			0.97
Hong et al. (42)	2023	ISIC-2017	0.94
Srikanteswara and Ramachandra (41)	2023		0.89
Rajendran and Shanmugam (52)	2024		0.97
Proposed model			0.98

Table 5 presents the results of the proposed model and those of the existing approaches, such as (38–41). The U-Net model was designed using skip paths to the encoder to reduce the semantic gap between concatenated maps of features for skin-lesion segmentation. The method was evaluated using PH2 and ISIC-18 datasets providing accuracies of 96.18 and 96.09%, respectively (38). The adaptive contour was applied for segmentation on the ISIC-17 and PH2 datasets, with accuracies of 0.94% and 0.96%, respectively (42). The LinkNet and U-Net models were combined to transfer skin-lesion segmentation learning on ISIC-18 and PH2 datasets with 0.89% and 0.92% accuracy (40). A two-stage method was designed based on a modified CNN classifier to segment the skin lesions. This method was evaluated on the PH2 and ISIC-17 datasets with accuracies of 0.82% and 0.89%, respectively.

### 4.3 Experiment 2: classification of the skin lesions using the proposed compact convolutional transformer model

**FIGURE 11:**
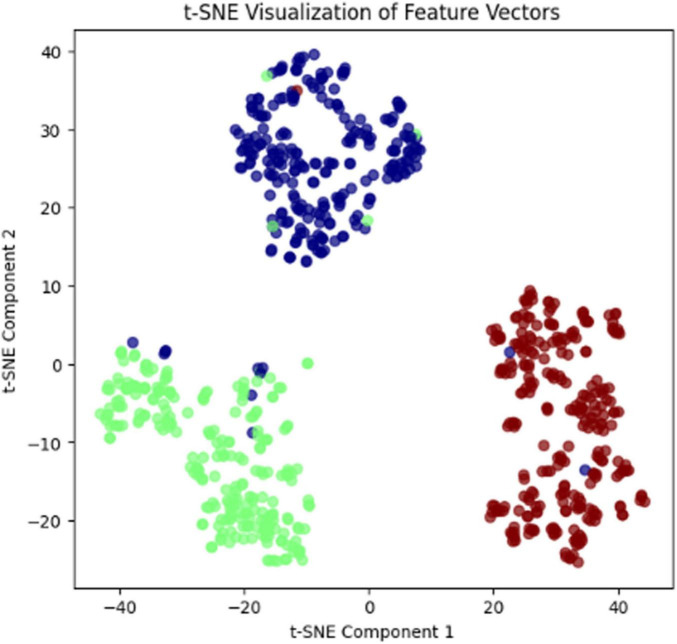
Features vector visualization for the PH2 dataset.

The proposed CCTM was trained for 100 epochs, and the results are shown in Table 6. The features vectors visualization for PH2 dataset is shown in Figure 11.

**TABLE 6:** Proposed classification results on the HAM-10000 dataset.

Akiec	Bcc	Bkl	Df	Mel	Nv	Vl	Precision (P)	Recall (R)	F1-score (F)
✓							0.96	0.96	0.96
	✓						0.96	0.97	0.96
		✓					0.95	0.95	0.95
			✓				0.98	1.00	0.99
				✓			0.96	0.93	0.95
					✓		0.95	0.96	0.96
						✓	1.00	1.00	1.00
✓	✓	✓	✓	✓	✓	✓	0.97 Accuracy (A)		
✓	✓	✓	✓	✓	✓	✓	0.97	0.97	0.97
✓	✓	✓	✓	✓	✓	✓	0.97	0.97	0.97

In Figure 11, t-SNE visualization of feature vectors illustrates how well the model has learned to distinguish between different classes. Each dot represents a sample in the dataset, and the color coding corresponds to different class labels. The clear separation of clusters indicates that the model has successfully extracted meaningful features, with samples of the same class grouping together while maintaining distinct boundaries between different classes. Some overlap might suggest areas where the model could improve, possibly due to similarities between certain categories. This visualization helps understand the model’s representation learning and provides insights into feature space organization.

**FIGURE 12:**
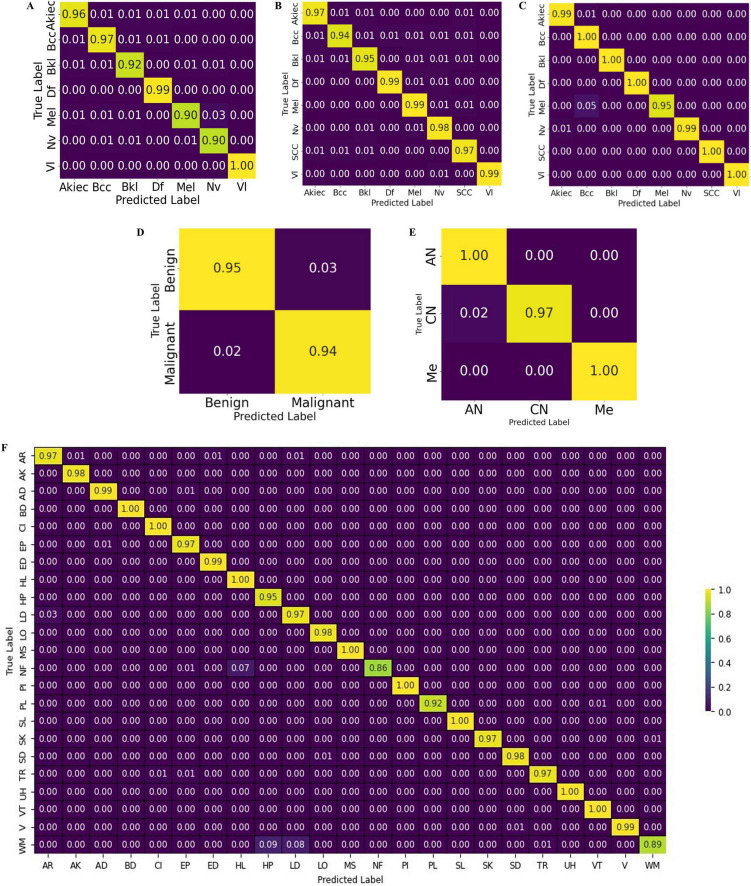
Confusion chart of the proposed CCTM for skin-lesion classification: **(A)** HAM-10000, **(B)** ISIC-2019, **(C)** ISIC-2020, **(D)** Med-node **(E)** PH2, and **(F)** DermNet.

The test results are shown in terms of the confusion matrix in Figure 12.

Table 6 presents the classification results on the HAM-10000 dataset. Precision, recall, and F1-score were computed for the individual classes, as well as the overall accuracy, micro average, and weighted average. The values of precision, recall and F1-score were 0.96, 0.96, and 0.96 for Akiec; 0.96, 0.97, and 0.96 for BCC; 0.95, 0.95, and 0.95 for Bkl; 0.98, 1.00, and 0.99 for Df; 0.96, 0.93, and 0.95 for Mel; 0.95, 0.96, and 0.96 for Nv; and 1.00, 1.00, and 1.00 for V1. The overall attained accuracy of the seven classes was 0.97, the macro average of the precision, recall, and F1 scores was 0.97, and the weighted average was 0.97.

**FIGURE 13:**
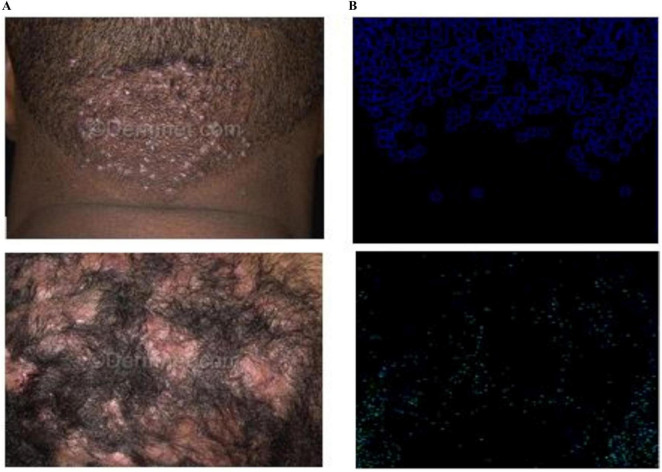
LIME results of the dark skin lesions: **(A)** input and **(B)** LIME.

The proposed CCTM classified all skin lesions, including light and dark lesions. Furthermore, to authenticate the model performance, explainable AI (XAI) was applied using LIME to highlight the important features of the model as shown in Figure 13.

The study addresses AI interpretability by applying explainable AI (XAI) techniques, specifically LIME, to highlight important features contributing to the model’s decisions. Figure 13 visually demonstrates how the proposed CCTM model classifies both light and dark skin lesions with transparency. This ensures clinical trust by making the model’s decision-making process more interpretable and justifiable.

The time elapsed was computed for both training and testing on the benchmark datasets, as shown in Table 7.

**TABLE 7:** Elapsed time on benchmark datasets.

Datasets	Execution time
HAM-10000	28 min 4 s
MED-NODE	15 min 2 s
ISIC-2019	23 min 1 s
ISIC-2020	21 min 0 s
PH2	16 min 0 s
DermNet	29 min 3 s

The running/execution time of the proposed model was also compared with that of the existing method (43), as listed in Table 8.

**TABLE 8:** Execution time comparison of the proposed CCTM model with the existing method.

Ref	Dataset	Running/execution time
Araújo et al. (43)	HAM-10000	1 h 30 min
Proposed Model	HAM-10000	28 min 4 s

Table 8 shows the elapsed time for the same benchmark dataset, where the execution time of the existing method is 1 h 30 min, whereas that of the proposed model is 28 min 4 s. Thus, the proposed model is computationally efficient. The classification outcomes are given in Table 9.

**TABLE 9:** CCTM classification outcomes using ISIC-2019 dataset.

Akiec	Bcc	Bkl	Df	Mel	Nv	Scc	Vl	P	R	F
✓								0.97	0.97	0.97
	✓							0.96	0.94	0.95
		✓						0.95	0.95	0.95
			✓					0.99	0.99	0.99
				✓				0.97	0.98	0.97
					✓			0.96	0.98	0.97
						✓		0.95	0.97	0.96
							✓	1.00	0.99	0.99
✓	✓	✓	✓	✓	✓		✓	0.97 A		
✓	✓	✓	✓	✓	✓		✓	0.97	0.97	0.97
✓	✓	✓	✓	✓	✓		✓	0.97	0.97	0.97

On ISIC-2019, the values of P, R, and F1 on the classes of Akiec were 0.97, 0.97, 0.97, whereas those of Bcc were 0.96, 0.94, 0.95, Bkl were 0.95, 0.95, 0.95, Df were 0.99, 0.99, 0.99, Mel were 0.97, 0.98, 0.97, Nv were 0.96, 0.98, 0.97, Scc were 0.95, 0.97, 0.96, and of VI were 1.00, 0.99, 0.99 respectively. The outcomes achieved for the ISIC-2020 dataset are listed in Table 10.

**TABLE 10:** CCTM classification outcomes using ISIC-2020 dataset.

Akiec	Bcc	Bkl	Df	Mel	Nv	Scc	Vl	P	R	F1
✓								0.99	0.99	0.99
	✓							0.95	1.00	0.97
		✓						1.00	1.00	1.00
			✓					1.00	1.00	1.00
				✓				1.00	0.95	0.97
					✓			1.00	0.99	1.00
						✓		1.00	1.00	1.00
							✓	1.00	1.00	1.00
✓	✓	✓	✓	✓	✓		✓	0.99 A		
✓	✓	✓	✓	✓	✓		✓	0.99	0.99	0.99
✓	✓	✓	✓	✓	✓		✓	0.99	0.99	0.99

Tables 10, 11 present the values of precision, recall, and F1-score on eight classes of ISIC-2020 dataset.

**TABLE 11:** CCTM classification outcomes using PH2 dataset.

Benign	Malignant	P	R	F
✓		0.97	0.96	0.97
	✓	0.96	0.97	0.97
✓	✓	0.97 A		
✓	✓	0.97	0.97	0.97
✓	✓	0.97	0.97	0.97

Table 11 presents results for benign and malignant classes. In the benign class, the results for P, R, and F1 were 0.97, 0.96, and 0.97, respectively, whereas those for the malignant class were 0.96, 0.97, and 0.97, respectively. Similarly, the accuracy was 0.97, and macro average and weighted rates were 0.97, 0.97, and 0.97 for precision, recall, and F1-scores, respectively. The classification results on MED-NODE dataset are listed in Table 12.

**TABLE 12:** CCTM classification outcomes using MED-NODE dataset.

AN	CN	Me	P	R	F1
✓			0.98	1.00	0.99
	✓		1.00	0.97	0.98
		✓	1.00	1.00	1.00
✓	✓	✓	0.99 A		
✓	✓	✓	0.99	0.99	0.99
✓	✓	✓	0.99	0.99	0.99

The classification results on the MED-NODE dataset were computed in three classes: AN, CN, and Me. The results of P, R, and F1 were 0.98, 1.00, and 0.99 for AN; 1.00, 0.97, and 0.98 for CN; and 1.00, 1.00, and 1.00 for the Me class, respectively. The accuracy was 0.99 and the macro-average and macro-weighted results were 0.99, 0.99, and 0.99, respectively. The results for the DermNet dataset are listed in Table 13.

**TABLE 13:** CCTM classification outcomes using DermNet dataset.

Classes	P	R	F1
AR	0.96	0.97	0.97
AK	0.99	0.98	0.99
AD	0.99	0.99	0.99
BD	0.99	1.00	1.00
CI	0.99	1.00	0.99
EP	0.97	0.97	0.97
ED	0.97	0.99	0.98
HL	0.93	1.00	0.96
HP	0.88	0.95	0.91
LD	0.98	0.97	0.98
LO	0.99	0.98	0.99
MS	0.99	1.00	1.00
NF	0.91	0.86	0.88
PI	1.00	1.00	1.00
PL	0.95	0.92	0.93
SL	1.00	1.00	1.00
SK	0.99	0.97	0.98
SD	0.98	0.98	0.98
TR	0.99	0.97	0.98
UH	1.00	1.00	1.00
VT	0.99	1.00	0.99
V	0.99	0.99	0.99
WM	0.93	0.89	0.91
Total Classes = 23	Accuracy = 0.97		
	0.97	0.97	0.97
	0.97	0.97	0.97

Table 13 provides results on twenty-three skin-lesion classes. On the classes the results of AR were 0.96, 0.97, 0.97, on AK were 0.99, 0.98, 0.99, whereas 0.99, 0.99, 0.99 on AD, 0.99, 1.00, 1.00 on BD, 0.99, 1.00, 0.99 on CI, 0.97, 0.97, 0.97 on EP, 0.97, 0.99, 0.98 on ED, 0.93, 1.00, 0.96 on HL, 0.88, 0.95, 0.91 on HP, 0.98, 0.97, 0.98 on LD, 0.99, 0.98, 0.99 on LO, 0.99, 1.00, 1.00 on Ms, 0.91, 0.86, 0.88 on Nf, 1.00, 1.00,1.00 on PI, 0.95, 0.92, 0.93 on PL, 1.00, 1.00, 1.00 on SL, 0.99, 0.97, 0.98 on SK, 0.98, 0.98, 0.98 on SD, 0.99, 0.97, 0.98 on TR, 1.00, 1.00,1.00 on UH, 0.99, 1.00,0.99 on VT, 0.99, 0.99, 0.99 on V and 0.93, 0.89, 0.91 on WM. The accuracy of all the classes is 0.97.

The CCTM outcomes validated using the ISIC-2019, ISIC-2020, PH2, and DermNet datasets are shown in Tables 5–9. The proposed model has an accuracy of 0.97 on ISIC-2019, 0.99 on ISIC-2020, 0.97 on PH2, 0.99 on MED-NODE, and 0.97 on DermNet datasets.

The CCTM model provides the highest accuracy of 0.99 on ISIC-2020 and MED-NODE compared with the other datasets. The CCTM results were compared with those of the existing methods, as presented in Table 14.

**TABLE 14:** Classification results of CCTM compared with those of existing methods.

References	Year	Datasets	Accuracy (%)
Rokhsati et al. (44)	2024	MED-NODE	0.96
Georgiadis et al. (53)	2025		0.66
Rasel et al. (54)	2024		0.75 F1
Proposed model			0.99
Rokhsati et al. (44)	2024	PH2	0.96
Reis et al. (39)	2024		0.88
Mustafa et al. (50)	2025		0.94
Georgiadis et al. (53)	2025		0.50
Proposed model			0.97
Singh et al. (45)	2024	ISIC-2020	0.73
Georgiadis et al. (53)	2025		
Proposed model			0.99
Reis et al. (39)	2024	ISIC-2019	0.96
Mustafa et al. (50)	2025		0.93
Georgiadis et al. (53)	2025		0.48
Proposed model			0.97
Singh et al. (45)	2024	HAM-10000	0.96
Mustafa et al. (50)	2025		0.92
Georgiadis et al. (53)	2025		0.73
Proposed Model			0.97
Medhat et al. (46)	2024	DermNet	91.92 ± 1.74
Mui-zzud-din et al. (47)	2024		0.91
Hanum et al. (55)	2025		0.94
Proposed model			0.97

Iterative magnitude pruning was used with AlexNet for skin-lesion classification on PH2 and MED-NODE datasets with an accuracy of 0.96 (44). The DT uses Bayesian learning and fuzzy ID3 values for skin-lesion classification. The results on PH2 and ISIC-19 datasets were 88% and 96%, respectively (39). A stacked CNN model was used for skin-lesion classification. This method was evaluated on ISIC-20 and HAM-10000 with accuracies of 0.73% and 0.96%, respectively (45). The features were optimized using GSO and the skin lesion was classified based on a random forest classifier (46). The ensemble model was created using a combination of pretrained models, such as ResNet50V2, ResNet152V2, and ResNet101V2, which were used for feature extraction to classify skin lesions (47).

In Table 14, on the MED-NODE dataset, where the proposed model achieved 99% accuracy, the misclassification rate is 1%. Similarly, for the PH2 dataset, achieving 97% accuracy, the misclassification rate is 3%. On the ISIC-2020 dataset, the proposed model outperformed previous methods with 99% accuracy, leading to a 1% misclassification rate. Likewise, for ISIC-2019, the model achieved 97% accuracy, corresponding to a 3% misclassification rate. On the HAM-10000 dataset, the proposed model attained 97% accuracy, resulting in a 3% misclassification rate. Finally, for the DermNet dataset, where the model achieved 97% accuracy, the misclassification rate remains 3%. These results indicate that the proposed model significantly reduces errors compared to previous studies while maintaining robust classification performance.

### 4.4 Ablation study

An ablation study was performed using both segmentation and classification models.

BASNet used ResNet-34 as a backbone because it gives a balance between performance and efficiency. It is a lightweight, less computationally expensive, and faster model. The residual connections and hierarchical features extraction abilities of this model help capture the contextual and fine-detail information. The pretrained weights of ResNet-34 enable faster training and better generalization. Compared with ResNet-50 and ResNet-101, deeper models exist, with higher computation and memory requirements and long training time.

Dermoscopy images of skin cancer often suffer from poor quality due to factors like lighting variations, hair occlusion, and low contrast, which can obscure critical features needed for accurate diagnosis. The hybrid loss and residual refinement alleviate the challenges of occlusion and poor contrast. Hybrid loss combines pixel-wise accuracy (e.g., cross-entropy) with structural sensitivity (e.g., dice loss), ensuring the model captures both fine details like lesion borders and broader patterns like texture irregularities. Residual refinement further improves predictions by iteratively correcting errors from earlier outputs, focusing on subtle but diagnostically significant features. This combination makes the system more robust and accurate, enabling it to handle the variability and imperfections common in dermoscopy images, ultimately supporting better skin cancer detection in real-world clinical applications. The hyperparameters of BASNet were finalized after the experimentation as shown in Table 15.

**TABLE 15:** Segmentation results on the variant of ablation using ISIC-2018 dataset.

Variant of ablation	Testing	Validation	Training	Average (%)	
				**IoU**	**Dice score**
Without using residual refinement	✓			0.78	0.79
		✓		0.75	0.76
			✓	0.75	0.78
Without using hybrid loss (structured similarity and boundary aware) Only binary cross-entropy	✓			0.79	0.76
		✓		0.77	0.75
			✓	0.78	0.77

Table 15 presents different types of losses and batch sizes. Learning rates and loss function were used for model training. In this experiment, an error rate of 0.049 was obtained on the combination of hyperparameters such as Hybrid loss, Adam optimizer, and le^−4^, which is less than those of others.

In BASNet model, weight (0.8) for pixel-wise (L2) loss and perceptual loss (0.2) were applied. This helped the model prioritize high-level preservation of features. The high weight on pixel-level loss, helps reconstruct the image and preserve the overall structure, particularly when fine details are less distinguishable due to occlusion or low contrast. This combination strikes a balance between fidelity and original ability to retain the vital features.

The segmentation BASNet model was authenticated by performing different experiments on the ISIC-2018 dataset, as listed in Table 16.

**TABLE 16:** Experiment for the selection of hyperparameters.

Batch size	Optimizer	Loss	Learning rate	Error rate
2	Sgdm	Tversky	le^−5^	0.093
***4***	***Adam***	***Hybrid***	***le* ^−4^**	***0.049***
8	RMSprops	Focal	le^−3^	0.100

The bold numbers represent the values selected from the experiment for further analysis and experimentation.

Table 16 presents the ablation variant without using the residual refinement module. The IoU was 0.78 and Dice score was 0.79 in the testing stage, the IoU was 0.75 and Dice score was 0.76 in the validation stage, and the IoU was 0.75 and Dice score was 0.78 in the training stage. Similarly, without using hybrid loss in the testing stage, IoU and Dice scores were 0.79 and 0.76, respectively. In the validation stage, IoU and Dice scores were 0.77 and 0.75, respectively. In the training stage, IoU and Dice scores were 0.78 and 0.77, respectively. It was observed that the residual refinement module and hybrid loss played vital roles in the segmentation of skin lesions. The results of segmentation drastically decrease when used without these parameters.

**FIGURE 14:**
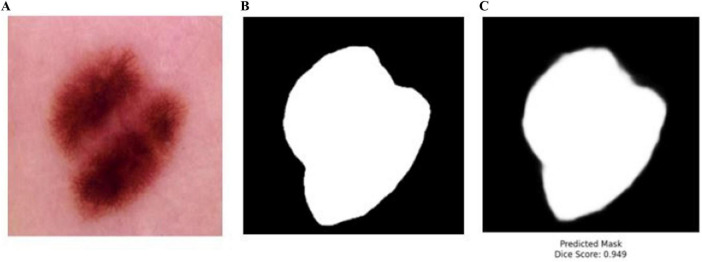
Testing of the BASNet on HAM-10000 dataset **(A)** input images **(B)** ground mask **(C)** predicted masks.

The proposed BASNet model was trained and tested on four benchmark datasets: PH2, ISIC 2016, ISIC 2017 and ISIC 2018. To authenticate the performance of BASNet, some images of the HAM-10000 dataset were passed to the trained weights on ISIC 2018 datasets; the segmentation results of the predicted masks with DSC scores are shown in Figure 14.

In Figure 14, BASNet was not trained on the BASNet model. The trained weights of BASNet on ISIS-2018 dataset were used to test some images of the HAM-10000 dataset. BASNet achieved 0.949 DSC, which shows the generalizability and reliability of the model. The training time of BASNet was compared with that of the existing method (Table 17).

**TABLE 17:** Comparison of training time.

Model	Time of each epoch
BASNet model	4 min
Öztürk and Özkaya (56)	432.3 sec

The training time of each epoch of BASNet is 4 minutes while that of the existing method, the iFCN model, is 432.3 seconds (Table 17). Before training CCTM, hyperparameters were selected after experimentation as shown in Table 18.

**TABLE 18:** Training hyperparameters of CCTM.

Conv layers	Project dimension	Number of heads	Transformer layers	Stochastic rate	Learning rate	Batch size	Error rate
***2***	***128***	***2***	***2***	***0.10***	***0.001***	***128***	***0.021***
4	64	4	4	0.01	0.01	64	0.054

The bold numbers represent the values selected from the experiment for further analysis and experimentation.

The hyperparameters which provide less error rates compared to others are highlighted in bold and italics, in Table 18. The results of the classification model were evaluated using a variant of ablation on the HAM-10000 dataset as shown in Table 19.

**TABLE 19:** Results of classification on variant of ablation using HAM-10000 dataset.

Variant of ablation	Akiec	Bcc	Bkl	Df	Mel	Nv	Vl	P	R	F1
Without convolution layers K	✓							0.82	0.82	0.80
		✓						0.82	0.81	0.81
			✓					0.80	0.79	0.85
				✓				0.81	0.80	0.78
					✓			0.82	0.83	0.74
						✓		0.78	0.79	0.74
							✓	0.80	0.81	0.79
✓	✓	✓	✓	✓	✓	✓	✓	0.80 Accuracy (A)		
✓	✓	✓	✓	✓	✓	✓	✓	0.80	0.80	0.80
✓	✓	✓	✓	✓	✓	✓	✓	0.80	0.80	0.80
Kernel size of convolution = 4	✓							0.90	0.91	0.90
		✓						0.91	0.90	0.92
			✓					0.90	0.89	0.90
				✓				0.91	0.92	0.91
					✓			0.92	0.93	0.90
						✓		0.92	0.92	0.92
							✓	0.91	0.91	0.91
✓	✓	✓	✓	✓	✓	✓	✓	0.90 A		
✓	✓	✓	✓	✓	✓	✓	✓	0.91	0.91	0.91
✓	✓	✓	✓	✓	✓	✓	✓	0.91	0.91	0.91
Without patch embedding	✓							0.81	0.80	0.89
		✓						0.80	0.81	0.82
			✓					0.80	0.81	0.82
				✓				0.82	0.82	0.82
					✓			0.81	0.80	0.80
						✓		0.80	0.81	0.80
							✓	0.81	0.81	0.81
✓	✓	✓	✓	✓	✓	✓	✓	0.82 A		
✓	✓	✓	✓	✓	✓	✓	✓	0.82	0.82	0.82
✓	✓	✓	✓	✓	✓	✓	✓	0.82	0.82	0.82

The results in Table 19 were computed based on different parameters, such as without convolution layers, by varying convolution kernel size, and without a patch embedding layer. Without convolutional layers, the accuracy was 0.80, whereas with the four kernel sizes of the convolutional layers, an accuracy of 0.90 was achieved. Similarly, without the patch embedding layer, an accuracy of 0.82 was achieved. The results can be drastically changed by reducing or changing the number of parameters.

## 5 Conclusion

Several studies have been conducted on the detection of skin lesions; however, accurate segmentation and classification of skin lesions remain great challenges. To overcome these challenges, we proposed a method, which is based on two novel models. To address the challenges of skin-lesion segmentation, a boundary-aware segmentation model was proposed based on hybrid loss and selected hyperparameters for more accurate skin-lesion segmentation. The model was assessed using four challenging dermoscopic datasets: PH2, ISIC-2016, ISIC-2017, and ISIC-2018. The average IoU and Dice scores were 0.96 and 0.98 for PH2; 0.89 and 0.96 for ISIC 2016; 0.94 and 0.97 for ISIC 2018; and 0.97 and 0.98 for ISIC 2017 datasets, respectively.

Skin-lesion classification remains a challenge owing to the similar shape, color, and size of skin lesions. Therefore, a CCTM was proposed and trained on optimal hyperparameters, achieving accurate skin-lesion classification at the testing stage. CCTM was evaluated on the ISIC Challenge and DermNet datasets with different types of skin lesions. The accuracy obtained was 0.99 on MED-NODE, 0.97 on PH2, 0.97 on ISIC-2019; 0.99 on ISIC-2020; 0.97 on HAM-10000, and 0.97 on DermNet datasets, respectively.

## 6. Limitations and future scope

BASNet is appropriate for poorly contrasted, illuminated, and hair dermoscopic images, it is computationally intensive. It focuses on both global and fine-grained details by employing a deep-learning model that undergoes several rounds of feature extraction, refinement, and fusion. This leads to significant processing and memory requirements, particularly when handling high-resolution dermoscopic images. Furthermore, the results demonstrated the superiority of CCTM. This is a great contribution to this domain; in the future, this model will be implemented in hospitals to evaluate its performance on real dermoscopic images. However, there remain obstacles to its incorporation into clinical workflows, including the need for strong regulatory approvals to guarantee safety, huge computing resources required for real-time inference, and the need for clinician training to properly understand AI results. For smooth adoption and for AI to support human knowledge in managing skin cancer rather than replace it, these obstacles must be overcome and cooperation between AI developers and healthcare practitioners must be encouraged.

In the future, a method using quantum machine/DL may be proposed to achieve accurate and efficient outcomes. The proposed method may also be validated on ISIC Challenge-2024, which was not used in this study.

## Data Availability

The original contributions presented in the study are included in the article/supplementary material. The code associated with this article can be found at: https://www.javeriaamin.site/2025/02/skin-lesion-segmentation-using-boundary.html. Further inquiries can be directed to the corresponding author.

## Footnotes

1
https://www.kaggle.com/datasets/shubhamgoel27/dermnet
